# Sleep-dependent memory consolidation and accelerated forgetting

**DOI:** 10.1016/j.cortex.2014.02.009

**Published:** 2014-05

**Authors:** Kathryn E. Atherton, Anna C. Nobre, Adam Z. Zeman, Christopher R. Butler

**Affiliations:** a Department of Experimental Psychology, University of Oxford, Oxford, UK; b Oxford Centre for Human Brain Activity, University of Oxford, Oxford, UK; c Cognitive and Behavioural Neurology Research Group, University of Exeter Medical School, UK; d Nuffield Department of Clinical Neurosciences, University of Oxford, Oxford, UK

**Keywords:** Accelerated long-term forgetting, Transient epileptic amnesia, Memory, Consolidation, Sleep

## Abstract

Accelerated long-term forgetting (ALF) is a form of memory impairment in which learning and initial retention of information appear normal but subsequent forgetting is excessively rapid. ALF is most commonly associated with epilepsy and, in particular, a form of late-onset epilepsy called transient epileptic amnesia (TEA). ALF provides a novel opportunity to investigate post-encoding memory processes, such as consolidation. Sleep is implicated in the consolidation of memory in healthy people and a deficit in sleep-dependent memory consolidation has been proposed as an explanation for ALF. If this proposal were correct, then sleep would not benefit memory retention in people with ALF as much as in healthy people, and ALF might only be apparent when the retention interval contains sleep. To test this theory, we compared performance on a sleep-sensitive memory task over a night of sleep and a day of wakefulness. We found, contrary to the hypothesis, that sleep benefits memory retention in TEA patients with ALF and that this benefit is no smaller in magnitude than that seen in healthy controls. Indeed, the patients performed significantly more poorly than the controls only in the wake condition and not the sleep condition. Patients were matched to controls on learning rate, initial retention, and the effect of time of day on cognitive performance. These results indicate that ALF is not caused by a disruption of sleep-dependent memory consolidation. Instead, ALF may be due to an encoding abnormality that goes undetected on behavioural assessments of learning, or by a deficit in memory consolidation processes that are not sleep-dependent.

## 1 Introduction

Memories are not static entities. Processes that occur after encoding alter memory traces and the likelihood that they will subsequently be successfully retrieved. Memory consolidation is a set of, as yet, poorly understood processes that transform initially labile memories into a more stable form (Stickgold & Walker, 2007). Neuropsychological models play a central role in the scientific study of human memory, but models of a pure consolidation deficit have thus far been conspicuously absent. This may be because the brain structures involved in memory consolidation overlap with those involved in memory encoding (Battaglia, Benchenane, Sirota, Pennartz, & Wiener, 2011; Mednick, Cai, Shuman, Anagnostaras, & Wixted, 2011). Patients with brain lesions affecting the long term retention of episodic memory therefore tend to have prominent learning deficits which confound the investigation of consolidation. Instead, a deficit in consolidation has been inferred from the ‘temporal gradient’ often seen in retrograde amnesia, whereby memories acquired shortly before brain injury are more vulnerable than those acquired remotely (e.g., Alvarez & Squire, 1994). However, studies of retrograde amnesia inherently suffer from a lack of experimental control over the memories under investigation. Moreover, the existence of a temporal gradient in episodic memory is disputed (Nadel & Moscovitch, 1997). Ideally, therefore, a neuropsychological model of memory consolidation would exhibit normal learning performance but excessively rapid forgetting. In this paper, we sought to investigate the cause of rapid forgetting in such a model, focussing particularly upon the role of sleep in the memory impairment.

### 1.1 Accelerated long-term forgetting (ALF)

ALF is a recently described memory impairment in which new information appears to be learnt and initially retained normally but then forgotten at an accelerated rate over subsequent days (Bell & Giovagnoli, 2007; Butler & Zeman, 2008a). There is some evidence to suggest that ALF may be restricted to declarative memory, which is dependent on the medial temporal lobes (MTLs) (Deak, Stickgold, Pietras, Nelson, & Bubrick, 2011; Muhlert, Milton, Butler, & Zeman, 2010). It has been proposed that ALF reflects a deficit in memory consolidation (e.g., Kapur et al., 1997).

ALF is particularly common amongst patients with transient epileptic amnesia (TEA), a form of late-onset epilepsy (mean onset 62 years, Butler et al., 2007). In TEA, seizures manifest as brief (30–60 min), recurrent episodes of memory loss (Butler et al., 2007; Kapur, 1990; Zeman, Boniface, & Hodges, 1998) which are sometimes associated with other features of epilepsy, most often olfactory hallucinations. While TEA patients typically perform within the normal range on interictal (between seizure) neuropsychological tests (Butler et al., 2007), approximately 50% complain of ALF (Zeman & Butler, 2010). The amnesic attacks in TEA usually cease with the initiation of anti-epilepsy medication, but the memory complaints often persist (Zeman & Butler, 2010).

Several lines of evidence point to the seizure focus in TEA lying in the MTLs (Zeman & Butler, 2010): (i) The memory loss experienced during attacks is similar to that occurring in other MTL disorders, including lesions (Squire & Zola-Morgan, 1991) and transient global amnesia (Bartsch & Butler, 2013); (ii) electroencephalography (EEG) evidence, when available, suggests a temporal lobe focus (Butler et al., 2007; Zeman et al., 1998); (iii) The common seizure-related symptom of olfactory hallucinations most likely reflects epileptic activity spreading out from the MTL to the nearby piriform cortex (Zeman & Butler, 2010); (iv) While brain scans in individuals with TEA are usually clinically normal, there is focal MTL atrophy at the group level (Butler et al., 2009, 2013); (v) A patient scanned during a flurry of attacks was found to have high signal in the left hippocampus on a T2-weighted magnetic resonance (MR) scan and hypermetabolism in the same region on a positron emission tomography (PET) scan, both of which had resolved once the seizures had been successfully treated (Butler & Zeman, 2008b).

The neural basis of ALF is unknown. Structural brain abnormalities have been identified in patients with ALF (e.g., Butler et al., 2009, 2013; Malmgren & Thom, 2012), but these have not been found to correlate with ALF severity. TEA patients have subtle atrophy in the hippocampus, but while this correlates with performance on standard tests of anterograde memory (which typically test memory at only 30 min after encoding), it does not correlate with ALF (Butler et al., 2009).

### 1.2 Possible link between ALF and sleep

A number of observations suggest a relationship between ALF and sleep.

There is a widely documented reciprocal relationship between sleep and epilepsy. Sleep modulates epileptic activity; slow wave sleep, in particular, has often been shown to increase it (Bazil, 2000; Bazil & Walczak, 1997; Goncharova, Zaveri, Duckrow, Novotny, & Spencer, 2009; Kotagal, 2001; Mayanagi, 1977; Nazer & Dickson, 2009; Romcy-Pereira, Leite, & Garcia-Cairasco, 2009; Rossi, Colicchio, & Pola, 1984; Sammaritano, Gigli, & Gotman, 1991). In turn, epilepsy often disrupts sleep, both in terms of subjective sleep quality and objectively measured sleep architecture (Bazil, 2000; Derrt & Duncan, 2013; Kotagal, 2001; Matos, Andersen, do Valle, & Tufik, 2010).

The amnesic attacks of TEA often occur upon waking (approximately 70% of patients have attacks in this context, Zeman & Butler, 2010), indicating that seizure activity may preferentially occur during sleep or at the transition from sleep to wakefulness (Butler et al., 2007). Further, TEA patients are more likely to show epileptiform abnormalities on sleep or sleep-deprived EEGs than wake EEGs (Butler et al., 2007; Zeman et al., 1998). And finally, ALF has been reported at delays as short as 24 h (i.e., after the first post-learning night of sleep) in groups of patients who have been shown to learn and initially retain new information normally (Fitzgerald, Thayer, Mohemed, & Miller, 2013; Martin et al., 1991; Muhlert et al., 2010).

### 1.3 Sleep and memory consolidation

There is now a large body of literature supporting the notion that sleep plays a major role in memory consolidation. The most prominent theory regarding the mechanism is that recently acquired representations are preferentially reactivated during sleep. In the context of declarative memories, this involves reactivations of memories in the MTLs during slow wave sleep (e.g., Peigneux et al., 2004; Wilson & McNaughton, 1994), which strengthen and stabilise the memories, making them more resistant to interference (Diekelmann, Büchel, Born, & Rasch, 2011) and more likely to be retained (Rasch, Büchel, Gais, & Born, 2007; Rudoy, Voss, Westerberg, & Paller, 2009).

### 1.4 Hypothesis

Subclinical epilepsy-related activity during sleep could disrupt the consolidation process. Studies in experimental animals support this idea: suppression of hippocampal sharp-wave ripples (which are associated with memory reactivations) using electrical pulses during post-learning sleep leads to impairments on spatial memory tasks (Girardeau, Benchenane, Wiener, Buzsaki, & Zugaro, 2009); and electrical induction of interictal spikes in the hippocampus during sleep impairs memory (Shatskikh, Raghavendra, Zhao, Cui, & Holmes, 2006).

A disruption of sleep-dependent memory consolidation has been commonly posited as a likely neurological basis of ALF (e.g., Butler et al., 2009; Holmes & Lenck-Santinin, 2006; Jansari, Davis, McGibbon, Firminger, & Kapur, 2010; Muhlert et al., 2011; Sud et al., 2014; Tramoni et al., 2011; Urbain, Di Vincenzo, Peigneux, & Van Bogaert, 2011; Zeman, Butler, Muhlert, & Milton, 2013). We sought to test this hypothesis. We compared retention over a night of sleep to that over a day of wakefulness in patients with TEA-associated ALF and healthy controls to determine whether the benefit of sleep for memory was reduced in these patients.

## 2 Methods

Before completing the main experiment comparing individuals with TEA-associated ALF and their matched controls, we ran a pilot study to ensure that the task was sensitive to the benefits of sleep and not confounded by circadian factors.

### 2.1 Pilot experiment

We used a word-pair associates task, which is declarative and MTL-dependent (Jackson & Schacter, 2004) and therefore likely to be affected by ALF. As our aim was to determine whether the benefit of sleep for memory is reduced in ALF patients, it was important to us to have a task that was sensitive to the benefit of sleep for memory in healthy people. Word-pair associates are the most commonly used declarative memory task in the sleep-dependent consolidation literature (Diekelmann & Born, 2010). Furthermore, if interference learning is introduced prior to testing, the benefit of sleep for memory consolidation can be demonstrated much more clearly, because sleep boosts memory's resistance to subsequent interference (Ellenbogen, Hulbert, Jiang, & Stickgold, 2009; Ellenbogen, Hulbert, Stickgold, Dinges, & Thompson-Schill, 2006). However, these studies have usually been carried out with young adults. The healthy participants for our main experiment would need to be matched in terms of age with the patients and so would all be over 50 years old. It has been proposed that there might be a decline in declarative memory consolidation during sleep with age (e.g., Backhaus et al., 2007). Therefore, we piloted our task to ensure that we had a robust sleep effect in healthy over 50s before embarking on the patient study.

#### 2.1.1 Participants

We tested eight healthy participants in our pilot study. Four of the participants were male. The mean age (±standard error of the mean – SEM) was 59 ± 1.65 years.

#### 2.1.2 Stimuli

The word-stimuli were nouns drawn from the MRC psycholinguistic database (Coltheart, 1981) with 4–7 letters, familiarity scores of 350–700, concreteness scores of 350–700, imagibility scores of 350–700, and British National Corpus frequency scores of 800–4300. Words were not used if they were emotionally potent or highly semantically similar to another word in the set. The words were split into six lists (set 1 A_1_, B_1_, C_1_ and set 2 A_2_, B_2_, C_2_) that were not significantly different from each other in terms of any of the aforementioned variables. Word-pairs, which were consistent across subjects, were made by combining the lists within the two sets. Words with obvious semantic relationships were not paired with each other.

#### 2.1.3 Task

Our paradigm had a sleep condition, in which participants were trained in the evening and tested in the morning (after a night of sleep) and a wake condition, in which participants were trained in the morning and tested in the evening (after a day of wakefulness, during which the participants were not permitted to nap). Stimuli were presented using Presentation (Neurobehavioral Systems, Albany, NY). The initial learning stage of the task involved training the participants to 60% criterion on 30 pairs of unrelated nouns (A–B). Memory performance was assessed using cued recall tests without immediate feedback, in which the participants were presented with the A words only and asked to produce the B words. Until they reached criterion on one of these tests, the participants were repeatedly re-exposed to the full set of pairs, with coloured borders around each pair (green for correct and red for incorrect) providing feedback on their most recent response.

Thirty minutes after the participants reached criterion, their memory was tested again. Twelve hours after the beginning of A–B pair training, following a retention interval of a night of sleep or a day of wakefulness, the participants were trained (with only one exposure) and immediately tested on 30 A–C interference pairs. These new pairs had the same cue words as the original pairs, but different paired associates. After a 10-min interval the participants were presented with the A words only and asked to produce both the B and C words. While we were primarily interested in the performance on the A–B pairs, we asked for the C paired associates as well in order to minimize retrieval competition (Ellenbogen et al., 2006, 2009). More details on the procedure can be found in the Supplementary material. The A–B and A–C stimulus sets contained four additional word-pairs each (two at the start and two at the end), which were used to buffer primacy and recency effects and were excluded from analyses.

In contrast to Ellenbogen's experiments (2006, 2009), on which our paradigm was based, we used a within-subjects design (to reduce the number of patients that would be required in the main experiment); everyone took part in both the sleep and wake conditions, with 24 h in between. Two different sets of A–B and A–C word-pairs were used, so that the stimuli were novel in each condition. The order of the conditions and the distribution of stimuli across conditions were counterbalanced across participants.

The test sessions were performed in a laboratory. For the pilot, the participants were allowed to go home to sleep and testing start times varied between seven and nine o'clock but were consistent for each participant.

### 2.2 Main experiment

#### 2.2.1 Participants

Thirteen patients, who met the diagnostic criteria for TEA, performed within the normal range on standard neuropsychological tests and reported symptoms suggestive of ALF, were recruited. The diagnostic criteria for TEA (taken from Zeman & Butler, 2010) were:

(1) A history of recurrent witnessed episodes of transient amnesia
(2) Cognitive functions other than memory judged to be intact during typical episodes by a reliable witness
(3) Evidence for a diagnosis of epilepsy based on one or more of the following:
  (a) Epileptiform abnormalities on EEG
  (b) The concurrent onset of other clinical features of epilepsy (e.g., lip-smacking, olfactory hallucinations)
  (c) A clear-cut response to anticonvulsant therapy.

Fifteen control participants were recruited by advertisement. To reduce the likelihood that the advertisements would attract people with concerns about their memory or sleep, these advertisements simply appealed for volunteers for psychology experiments and did not specify the cognitive functions under investigation. One control did not reach criterion on the task and did not complete the experiment. A control was excluded from analyses because he was an outlier on the 12 h test (his performance was more than two standard deviations below the mean of the other participants). A further three participants were excluded. The youngest control participant was removed to make the patient and control groups more closely matched in terms of age. One patient repeatedly delayed the experimental proceedings, meaning that he took an unusually long time over the training procedure (his average response time in the training tests was more than four standard deviations greater than the mean of the included participants), and the other was removed to allow better counterbalancing of the versions of the experiment. Exclusion of these three participants did not alter the significance of any of the long-term memory retention results we report [though it should be noted that, if these participants had been included, the patients would have been found to perform significantly worse than the controls overall on the word-pair associates memory tests (i.e., a main effect of group in the first analysis of variance (ANOVA) reported in Section 3.2.1), rather than just numerically worse, with *p* = .062, see Section 3.2.1]. However, if they had not been excluded, the two groups would not have been matched in terms of age, experiment version and performance over the first 30 min of the experiment, which could have confounded interpretation of the results.

The 11 remaining patients (see Table 1) were matched in terms of age, IQ and performance on a range of standard neuropsychological tests (see Table 2) to the group of 12 remaining control participants. All patients were on anticonvulsant monotherapy and had been free of any clear-cut amnesic attacks or other seizures for at least 6 months prior to testing. The control participants did not suffer from any psychiatric, central nervous or sleep disorders and did not complain of ALF. The participants did not do shift work, did not consume alcohol during the experiment, and had not crossed time zones in the preceding weeks.

The study received ethical approval from the Scotland A Research Ethics Committee and written informed consent was obtained from all participants.

#### 2.2.2 Tasks

##### 2.2.2.1 Word-pairs

**Fig. 1:**
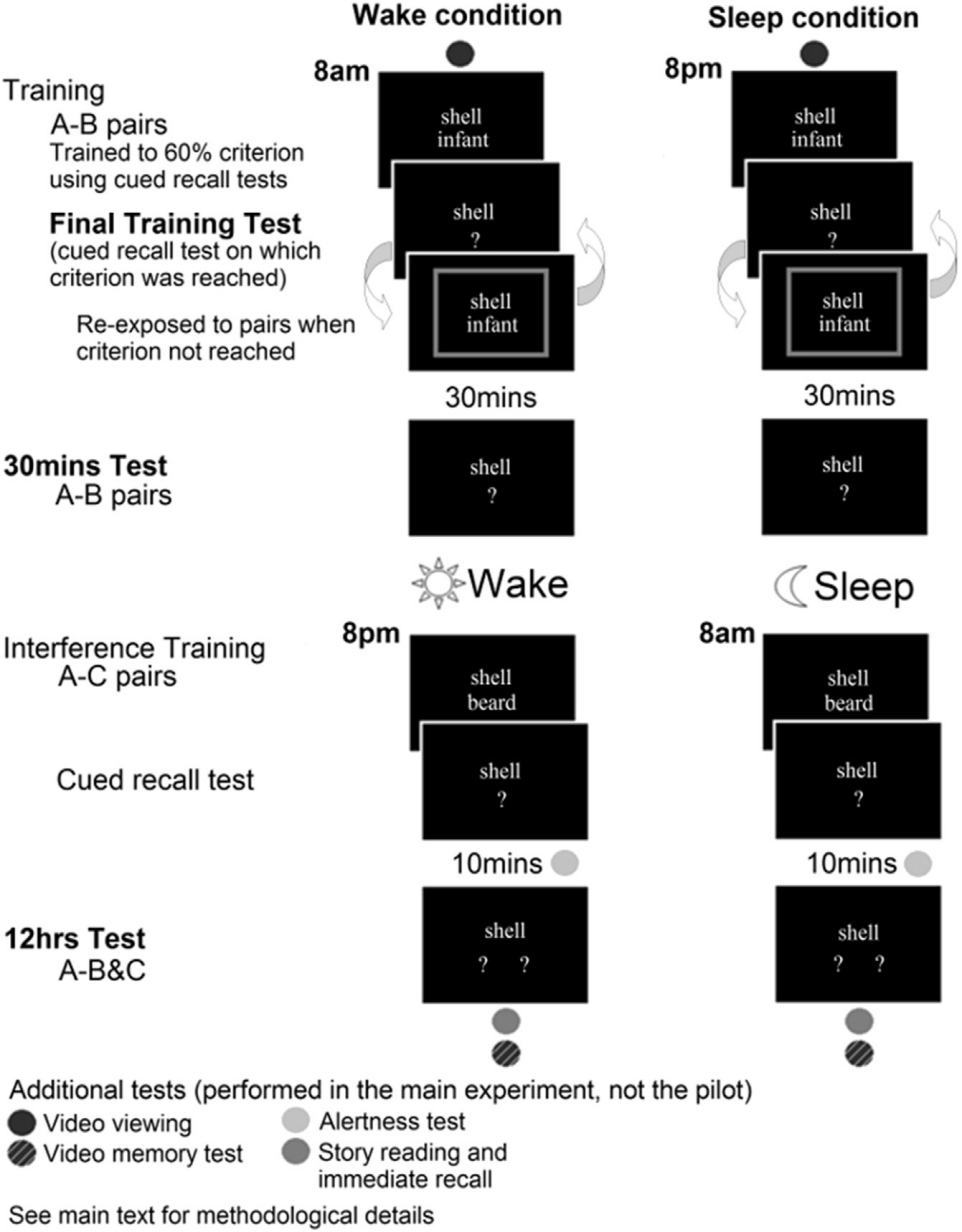
An illustration of the word-pair associates paradigm. Each participant took part in both the sleep and wake conditions, with 24 h in between. Two word-pair sets were used in the experiment so that the stimuli were novel in each condition (examples from only one of the word-pair sets are shown in the figure). The order of the conditions and the distribution of stimuli across conditions were counterbalanced across participants. Additional tests (represented by circles and detailed in Section 2.2.2.2) appear on the schematic to illustrate the order of events. These additional tests were performed in the main experiment only, and not in the pilot experiment.

The procedure for the word-pair task was the same as that used in the pilot study. See Fig. 1 for an illustration of the procedure. The A–B pair training began at approximately eight o'clock (in the morning in the wake condition, and in the evening in the sleep condition) and A–C pair training began 12 h later. All training and test sessions took place in our sleep laboratory. The participants slept in the sleep laboratory during the sleep condition. Every participant had an adaptation night of sleep in the laboratory prior to their sleep condition.

For the main analysis, we performed a mixed-effects ANOVA with A–B pair performance as the dependent variable, sleep condition (two levels: sleep and wake) and retrieval time point (three levels: final training test, 30 min test and 12 h test) as the within-subjects factors and group as the between-subjects factor.

##### 2.2.2.2 Subsidiary tasks

###### 2.2.2.2.1 Video

We ran a video-based memory test during the same experimental sessions. This task was intended to provide a more naturalistic memory test, involving moving images of people, sounds and dialogue. The procedure involved showing the participants a short film (just prior to A–B pair training). They were instructed to pay close attention to the film and were informed that their memory would be tested later. Approximately 12 h later (shortly after the 12 h word-pair test), after a night of sleep or a day of wakefulness, the participants were reminded that they had been shown a short film 12 h earlier, and were asked to recall the chain of events that happened in the film, not missing anything out if possible. The two films that we used for this experiment were dramatised short stories and they were counterbalanced across the sleep and wake conditions. They were of similar length (approximately 3 min each) and were both made by the same local film company. Prior to the experiment, we identified the plot points in each film. The participants were scored according to the percentage of the plot points they successfully recalled 12 h after viewing the film. Given that TEA patients with ALF have been shown to forget real-life events at an accelerated rate (Muhlert et al., 2010), one might expect patients to perform more poorly than controls on this task. However, since it involved neither a baseline memory assessment (i.e., there was no test prior to the night of sleep/day of wakefulness) nor interference learning, we were not confident that a benefit of sleep for memory performance would be detected in this task. The data were arcsine transformed for the purpose of statistical tests, but the means and SEMs are reported in untransformed form. The data were analysed using a mixed-effects ANOVA with recall performance as the dependent variable, sleep condition (sleep and wake) as the within-subjects factor and group as the between-subjects factor.

The data from one of the patients had to be excluded because he was not wearing his hearing aid while viewing one of the films, and this may have contributed to his poor recall results. Two of the control subjects did not participate in the video test.

###### 2.2.2.2.2 Alertness

The alertness task was designed to test the participants' reaction times in the morning and evening and thereby provide a circadian control for the word-pair experiment. The task was performed during the 10-min interval between A–C pair training and the 12 h word-pair tests. The experiment was implemented in Presentation (Neurobehavioral Systems, Albany, NY). Two white circles (3.1 cm in diameter) were presented side by side in the centre of the screen (.9 cm apart) against a black background for the duration of the experiment. Each circle was associated with a particular button on the keyboard (‘x’ for the left circle and ‘,’ for the right circle, which were operated with the left and right index fingers, respectively). Whenever a white asterisk (1.6 cm in diameter) appeared in one of the two circles, the participant had to respond as quickly as possible with the corresponding button, at which point the asterisk would disappear. The inter-stimulus-interval varied unpredictably between .5 sec, 1 sec, 2 sec and 4 sec. Each participant performed the task twice: once in the evening just before the wake condition's 12 h word-pair test, and once in the morning, just before the sleep condition's 12 h word-pair test. The first ten trials were considered practice trials and were excluded from the analysis. The reaction times from the following 38 trials were analysed.

The data were analysed using a mixed-effects ANOVA with reaction time as the dependent variable, time of day (morning and evening) as the within-subjects factor and group as the between-subjects factor.

###### 2.2.2.2.3 Immediate story recall

The immediate story recall task was designed to test the participants' memory processes in the morning and evening and thereby provide a circadian control for the word-pair experiment. Following each 12 h word-pair test, the participants were read a story. The stories we used were numbers 3 and 4 from the Birt Memory and Information Processing Battery tests (BMIPB; Coughlan, Oddy, & Crawford, 2007), and they were counterbalanced across conditions. The participants were instructed to pay close attention to the story because they would be asked to say it back to the experimenter shortly after hearing it. Once the experimenter had finished the story, the participants were asked to count back from 100 in 3s to prevent rehearsal of the story in working memory. After approximately 40 sec of this distraction, the participants were asked to recall the story they had just heard. Performance was scored according to the BMIPB criteria.

The data were analysed using a mixed-effects ANOVA with story recall score as the dependent variable, time of day (morning and evening) as the within-subjects factor and group as the between-subjects factor.

The data from two of the patients had to be excluded. One patient thought he had heard one of the stories before, and another had a severe emotional reaction to one of the stories because it reminded him of the circumstances surrounding his friend's death.

## 3 Results

### 3.1 Pilot study

**Fig. 2:**
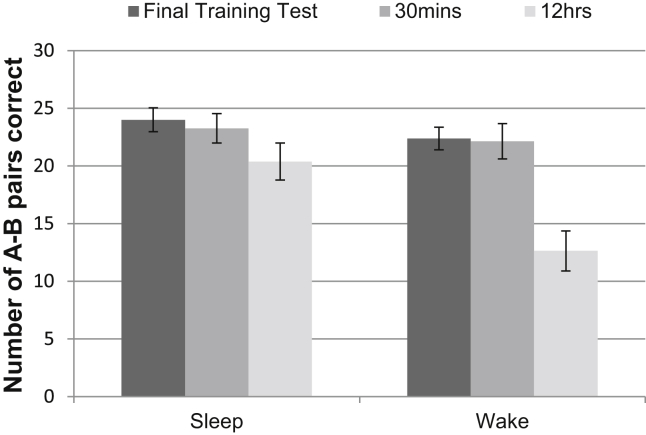
A–B pair performance on the final test of the training session, the 30 min test and the 12 h test in the sleep and wake conditions of the healthy older adults pilot. Error bars represent SEMs.

The pilot data clearly demonstrate a benefit of sleep versus wake for retrieval of paired associates. Fig. 2 plots the final training test, 30 min and 12 h A–B pair scores for the sleep and wake conditions. A repeated-measures ANOVA revealed not only a significant main effect of retrieval time point [*F*_(2,14)_ = 41.28, *p* < .001, with poorer performance on the 12 h test (estimated marginal mean: 16.50 ± 1.31) than the 30 min test (22.69 ± 1.17) (*p* = .001) and the final training test (23.19 ± .74) (*p* < .001)], but also a significant interaction between retrieval time point and sleep condition [*F*_(1.21,8.44)_ = 13.04, *p* = .005]. There was no significant difference between the sleep and wake conditions on the final training test (24.00 ± 1.04 and 22.38 ± .98, respectively, *p* = .28) or the 30 min test (23.30 ± 1.28 and 22.13 ± 1.53, respectively, *p* = .50), but the A–B pair score at 12 h was significantly greater in the sleep condition (20.38 ± 1.61) than the wake condition (12.63 ± 1.74) (*p* = .008).

A significantly greater percentage of pairs was lost between the 30 min and 12 h tests in the wake condition (42.68 ± 7.38%) than in sleep condition [12.82 ± 3.67%, *t*_(7)_ = −4.00, *p* = .005].

The conditions differed in terms of time of day of learning and testing as well as whether or not there was sleep in the interval. Notably, testing took place in the morning in the sleep condition but in the evening in the wake condition. However, performance in the learning phase (in the morning for the wake condition and in the evening for the sleep condition) was not significantly different between the conditions, making it unlikely that circadian factors could account for the superior performance in the sleep condition. There was no significant difference between sleep and wake conditions in score on the first training test (15.50 ± 3.03 and 14.63 ± 2.90, respectively, *p* = .57) or number of trials to criterion (1.88 ± .30 and 1.88 ± .30, *p* = 1.00). Furthermore, there was no significant difference between sleep and wake conditions in immediate interference pair score (14.75 ± 2.55 and 15.38 ± 2.24, respectively, *p* = .76) or interference pair score on the second test (13.75 ± 2.90 and 15.00 ± 2.26, respectively, *p* = .51).

### 3.2 Main experiment

#### 3.2.1 Word-pairs

**Fig. 3:**
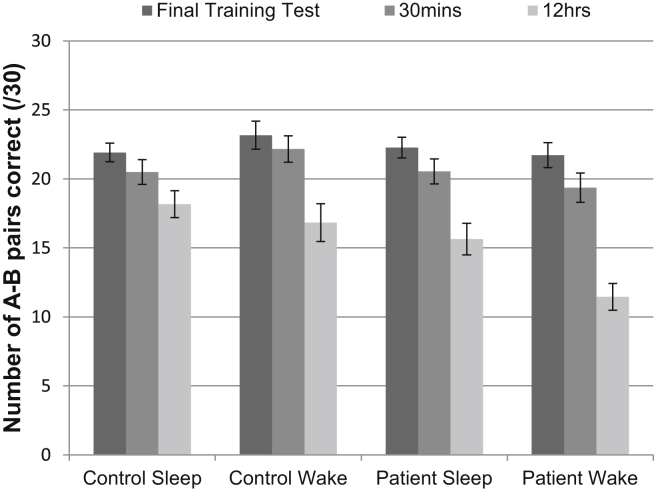
A–B pair performance across the three memory test sessions in the sleep and wake conditions of the word-pair associates task, in TEA patients with ALF and control participants. Error bars represent SEMs.

Table 3 contains the performance scores on the word-pair task. Fig. 3 displays the A–B performance scores on the final test of the training phase, and on the 30 min and 12 h tests in the sleep and wake conditions for the patient and control groups.

As expected, there was a significant main effect of retrieval time point [*F*_(1.34,28.15)_ = 99.24, *p* < .001]. Memory performance declined significantly between each test (*p* < .001 in all cases, estimated marginal means: 22.27 ± .47, 20.64 ± .53, 15.52 ± .70, for the final training test, 30 min test and 12 h test, respectively).

There was a significant interaction between retrieval time point and group [*F*_(1.34,28.15)_ = 6.33, *p* = .012]. The patients performed significantly worse than the controls on the 12 h test (estimated marginal means: 13.55 ± 1.01 and 17.50 ± .97, respectively, *p* = .01), but not on the final training test (22.00 ± .68 and 22.54 ± .65, *p* = .57) or the 30 min test (20.00 ± .76 and 21.33 ± .73, *p* = .21). The overall main effect of group was not quite significant (*p* = .062). This is in keeping with the typical profile of ALF: normal learning and initial retention but rapid forgetting over the longer-term.

There was a clear benefit of sleep for memory in this experiment; there was a significant interaction between sleep condition and retrieval time point [*F*_(2,42)_ = 20.15, *p* < .001]. The participants performed significantly better in the sleep condition than the wake condition on the 12 h test (estimated marginal means: 16.90 ± .75 and 14.14 ± .85, *p* = .002), but not on the final training test (22.10 ± .51 and 22.45 ± .68, *p* = .74) or the 30 min test (20.52 ± .64 and 20.77 ± .71, *p* = .85).

However, there was no significant interaction between sleep condition, retrieval time point and group (*p* = .55), meaning that the patients did not show a reduced benefit of sleep for memory compared to controls. Both groups showed a benefit of sleep for memory: there was a significant interaction between sleep condition and retrieval time point for both the patients [*F*_(1,10)_ = 7.73, *p* = .019] and the controls [*F*_(1,11)_ = 21.21, *p* = .001] when repeated-measures ANOVAs were performed separately for the two groups [with sleep condition (sleep and wake) and retrieval time point (30 min and 12 h) as factors]. In fact, the patients only performed significantly more poorly than the controls on the 12 h test in the wake condition [*t*_(21)_ = 3.16, *p* = .005] and not in the sleep condition [*t*_(21)_ = 1.69, *p* = .11], which is the reverse of what would be expected if ALF were caused by a disruption of sleep-dependent memory consolidation.

A mixed-effects ANOVA, with the percentage of A–B pairs lost between the 30 min and 12 h tests as the dependent variable, showed that more was forgotten in the wake condition (estimated marginal mean: 32.81 ± 2.84%) than the sleep condition (17.10 ± 3.52%) [*F*_(1,21)_ = 28.58, *p* < .001], and more was forgotten by the patients (32.03 ± 4.10%) than the controls (17.88 ± 3.93%) [*F*_(1,21)_ = 6.21, *p* = .021]. There was no interaction between sleep condition and group (*p* = .57), confirming that there was no reduction in the benefit of sleep for memory in the patients. In fact, patients only forgot significantly more than the controls in the wake condition [40.75 ± 4.11% *vs* 24.88 ± 3.93%, *t*_(21)_ = −2.79, *p* = .011] and not in the sleep condition [23.32 ± 5.08% *vs* 10.88 ± 4.86%, *t*_(21)_ = −1.77, *p* = .091].

There were more women in the control group than the patient group. However, this cannot account for our results. It is not the case that the difference between the patients and the controls on the 12 h test was due to the women in the control group performing particularly well (female control performance on the 12 h test in the wake condition: 16.60 ± 1.72, males: 17.00 ± 2.10; female control performance on the 12 h test across both conditions: 17.40 ± 1.51, males: 17.57 ± 1.52). It is also not the case that poor sleep-dependent memory consolidation in the female controls relative to the males obscured a true deficiency in sleep-dependent memory consolidation in the patients. The benefit of sleep for memory consolidation [measured in terms of the difference in percentage forgetting (between the 30 min and 12 h tests) between the wake and sleep conditions] in the male control participants alone (15.17 ± 5.27%), while slightly greater than the control group mean (14.00 ± 3.40%), was still numerically smaller than that in the patient group (16.93 ± 4.99%) i.e., the patients showed a numerically greater benefit of sleep for memory than the controls, and this was still true when only the results from the male controls were considered.

#### 3.2.2 Control tests

##### 3.2.2.1 Learning performance

The patients and controls did not differ in terms of learning performance. There was no significant difference between the patients and controls in terms of score on the first A–B pair training test in the sleep (*p* = .58) or wake (*p* = .20) conditions, or when the scores were collapsed across conditions (*p* = .35). The same was true for the trials to criterion (*p* = .17, *p* = .55 and *p* = .48).

##### 3.2.2.2 Circadian

###### 3.2.2.2.1 Learning performance

In this experiment, the two conditions did not differ only in terms of whether the retention interval contained sleep, but also in the time of day of training and testing. This provides a potential circadian confound, which could account for the apparent benefit of sleep for memory in our experiment i.e., if the participants generally performed better in the morning, this could explain their better performance on the 12 h test in the ‘sleep’ condition relative to the ‘wake’ condition. However, if this were true, then the participants would have performed better in the A–B training session in the wake condition than in the sleep condition, and this was not the case. As in our pilot study, participants did not perform significantly better in terms of trials to criterion (*p* = .62) or score on the first training test (*p* = .97) in the wake condition than the sleep condition.

Additional evidence that an effect of time of day on general performance levels cannot account for our results comes from the alertness test and the immediate story recall test, which were administered during the 12 h test sessions.

###### 3.2.2.2.2 Alertness

**Fig. 4:**
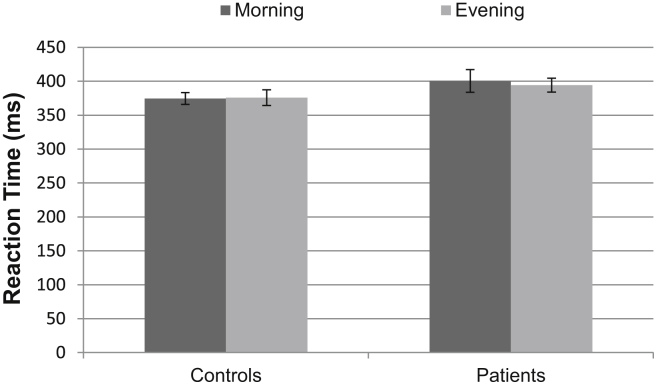
Reaction time data from the alertness test. Error bars represent SEMs.

The participants were not faster to respond in the morning than the evening (*p* = .64), suggesting that they were not more alert during the test session in the sleep condition than the wake condition. The patients' reaction times were not significantly different from those of the controls (*p* = .19), and there was no interaction effect between time of day and group (*p* = .47), suggesting that time of day was not differentially affecting performance in the two groups. See Fig. 4 for the reaction time data.

###### 3.2.2.2.3 Immediate story recall

**Fig. 5:**
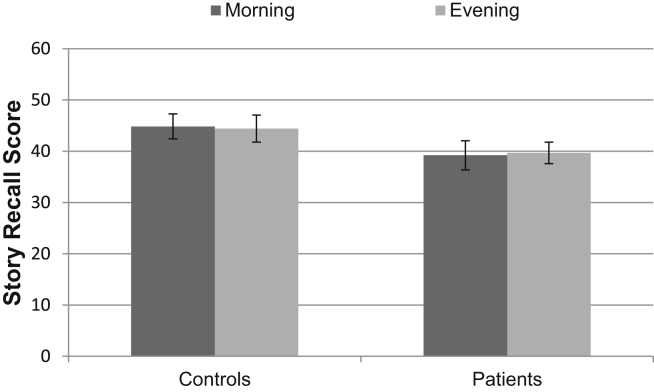
Retrieval performance on the immediate story recall task. Error bars represent SEMs.

Similarly, there was no effect of time of day on performance in the immediate story recall task (*p* = .99), no significant difference between the groups (*p* = .14) and no interaction effect (*p* = .77). See Fig. 5 for the immediate story recall data. This suggests that the benefit of sleep for memory in our experiment cannot be accounted for by the participants' memory retrieval systems simply functioning better in the morning than the evening. The lack of an interaction effect shows that a greater general cognitive decline in the patient group across the course of a day is not the explanation for the patient's poor 12 h word-pair test performance in the wake condition and normal performance in the sleep condition.

##### 3.2.2.3 Interference

Another possible confound for the benefit of sleep for memory would be differential A–C pair performance in the sleep and wake conditions, which would mean different levels of interference. However, A–C pair performance was not significantly different in the two conditions for the controls (*p* = .37) or patients (*p* = .25) or when collapsed across the two groups (*p* = .85).

If the patients had performed better than the controls on the A–C pairs, this would have provided a confound for the group differences in A–B pair performance on the 12 h test and forgetting rates between the 30 min and 12 h tests. However, A–C pair performance was not significantly different in the two groups for the wake condition (*p* = .56) or the sleep condition (*p* = .58) or when collapsed across the two conditions (*p* = .97).

**Fig. 6:**
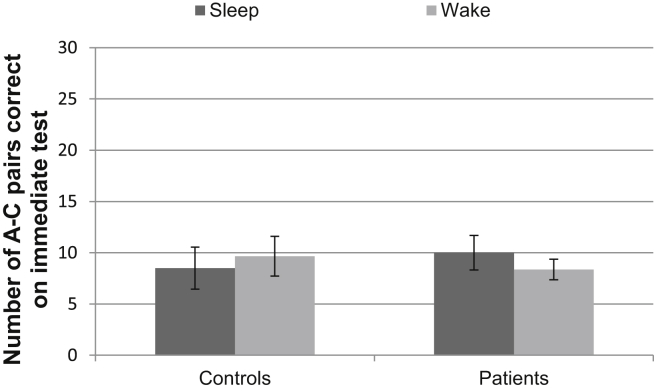
Performance on the immediate A–C pair test. Error bars represent SEMs.

See Fig. 6 for performance data on the immediate A–C pair test.

#### 3.2.3 Video task

**Fig. 7:**
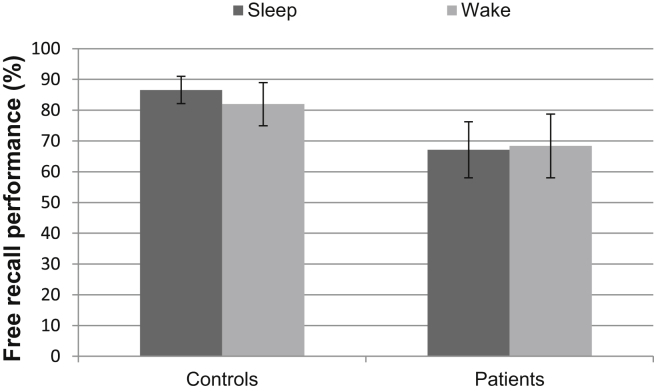
Free recall performance on the video memory test, which was administered approximately 12 h after the video was viewed. Error bars represent SEMs.

The patients were significantly worse than controls at recalling the content of a film 12 h after viewing it [*F*_(1,18)_ = 9.11, *p* = .007]. However, the task was apparently not preferentially consolidated by sleep – there was no effect of sleep condition in the ANOVA (*p* = .85), nor was there a significant difference between conditions for the healthy controls alone (*p* = .80) – and there was no interaction between sleep condition and group (*p* = .91), suggesting that sleep did not affect the two groups differently. See Fig. 7 for the video memory test data.

## 4 Discussion

This experiment was designed to test the frequently proposed hypothesis that ALF is caused by a disruption of sleep-dependent memory consolidation (e.g., Butler et al., 2009; Holmes & Lenck-Santinin, 2006; Jansari et al., 2010; Muhlert et al., 2011; Tramoni et al., 2011; Urbain et al., 2011; Zeman et al., 2013). We compared memory retention over a night of sleep and a day of wakefulness in TEA patients with ALF and control participants.

We first used a pilot study in a separate group of healthy participants to establish that our word-pair associates paradigm was sensitive to the benefit of sleep for memory retention in healthy older adults. This meant that it was a suitable instrument with which to test for a reduced benefit of sleep for memory in our patient group.

In sharp contrast to our expectations, we found that sleep boosted memory retention in the TEA ALF patients and that this sleep benefit was equivalent in the patients and in the controls. In fact, if sleep disruption were the cause of ALF, then one might expect patients to only show a memory impairment when the retention interval contains sleep. This was not the case. Indeed, the opposite was found: the patients showed a significant memory impairment relative to controls in the wake condition only, and not in the sleep condition. This suggests that sleep is not causing or exacerbating the memory problem in these patients. If anything, it may protect them from it in some cases.

These results are in line with a pilot study in temporal lobe epilepsy (Deak et al., 2011) in which patients forgot significantly more than controls over a day of wakefulness but not over a night of sleep. This pilot study did not fully control for potential group differences in learning and circadian fluctuations in performance, and the task was not sensitive to the benefit of sleep for memory consolidation in healthy control subjects. Therefore, our findings, in a different and larger group of patients, add weight to their conclusion that ALF is not caused by a deficit in sleep-dependent memory consolidation. Our results are also compatible with those of Fitzgerald et al. (2013), who found no relationship between night-time sleep architecture (recorded with EEG) and ALF in epilepsy patients.

We also report the novel finding that ALF in TEA can be seen across an interval as short as 12 h. Twenty-four hours is the shortest interval over which ALF in TEA has been reported to date (Muhlert et al., 2010), which contributed to the conjecture that memory loss was mediated by sleep (Muhlert et al., 2011). We found poorer performance in the patients than the controls 12 h after encoding for both word-pair associates with interference learning and for more naturalistic video-based stimuli. In contrast to the word-pair associates task, the video test was not sensitive to the benefit of sleep for memory retention. One reason for this may be that the task did not involve interference learning.

It is worth noting that both our pilot study and our main experiment demonstrate that sleep is still beneficial for memory in older people. The vast majority of sleep-dependent memory consolidation experiments have used only young healthy participants, and it has been suggested that sleep-dependent memory consolidation may decline with age (e.g., Backhaus et al., 2007; Peters, Ray, Smith, & Smith, 2008; Spencer, Gouw, & Ivry, 2007; Wilson, Baran, Pace-Schott, Ivry, & Spencer, 2012). However, our finding that sleep still benefits memory in some tasks in older adults is in line with some recent studies from other laboratories (Aly & Moscovitch, 2010; Tucker, McKinley, & Stickgold, 2011; Wilson et al., 2012).

The TEA patients in our study forgot at an accelerated rate after apparently normal learning and initial retention, but did not show a reduced benefit of sleep for memory. If not disrupted sleep-dependent memory consolidation, then what is the cause of ALF? One possible explanation is that the patients actually suffer from a subtle encoding deficit that goes undetected during learning and early retention, but ultimately leads to accelerated forgetting. This seems unlikely, as some researchers have gone to great efforts to match patients and controls for learning and initial retention and still demonstrate ALF over the longer-term (e.g., Hoefeijzers, Dewar, Della Sala, Zeman, & Butler, 2013). However, it remains possible that these behavioural measures were not sufficiently sensitive, and it would be interesting to study encoding-related brain activity in ALF patients.

Alternatively, ALF may be caused by a disruption of consolidation processes that are not sleep-specific. The processes that are disrupted in ALF patients might primarily occur during the waking state in healthy people. It may be that ALF patients struggle to cope with the competing demands of consolidating recently-acquired information and processing new information, making them particularly susceptible to the interference from ongoing cognitive processes that occur during waking hours. Temporal lobe amnesics have been shown to be particularly vulnerable to interference immediately after encoding (Dewar, Garcia, Cowan, & Sala, 2009). Unfortunately, our design cannot be used to investigate susceptibility to interference because, due to power concerns, all of our A–B pairs were interfered with. Another possibility is that patients with ALF are unable to consolidate normally during rest while awake, as healthy people do (e.g., Mednick, Makovski, Cai, & Jiang, 2009; Tambini, Ketz, & Davachi, 2010).

A consolidation deficit in ALF patients could be caused by structural or functional brain abnormalities. Some structural brain imaging studies have been done in patients with ALF, and while abnormalities have been found, notably in the MTL (e.g., Butler et al., 2007), a structural correlate of ALF has not been identified (Butler et al., 2013). However, it is conceivable that the methods used were not sufficiently sensitive. Furthermore, studies to date have focused on regions of grey matter, and it is possible that ALF patients suffer from abnormalities in structural connectivity between brain areas. Epilepsy is characterised by the aberrant propagation and synchronisation of electrical activity through neural connections. Abnormal structural connectivity could be a contributing factor to, or a consequence of, the epilepsy that is associated with ALF. Neural network dynamics are thought to play a critical role in systems consolidation (e.g., Battaglia et al., 2011; Tambini et al., 2010), and so abnormal structural connectivity could lead to a consolidation deficit. Even if structural abnormalities cannot be detected, it may be the case that the functional connectivity on which consolidation depends is abnormal in ALF patients. Precisely-timed communication between the hippocampus and the neocortex is thought to underlie declarative memory consolidation (e.g., Battaglia et al., 2011; Ji & Wilson, 2007), and this may be disrupted in ALF patients. One possible source of disruption would be epileptiform activity. The patients in our study were seizure-free, but interictal discharges could have occurred during our experiment, and some studies have found a relationship between ALF and electrical discharges in the retention interval in patients with epilepsy (e.g., Fitzgerald et al., 2013).

Other possible explanations for ALF include the effects of anti-epileptic medications and psychosocial factors. However, we consider these to be unlikely explanations. Complaints of ALF in TEA typically pre-date the onset of treatment (Butler et al., 2007; Butler & Zeman, 2008a), the patients are on low doses of medication, and they often report some degree of memory improvement once they begin treatment (Butler et al., 2007; Gallassi, 2006; Gallassi, Morreale, Lorusso, Pazzaglia, & Lugaresi, 1988; Zeman et al., 1998). Mood disorders do not clearly impair long-term memory retention. Lewis and Kopelman (1998) found that depressed patients did not forget more than controls, once initial learning was equated. Furthermore, our patients did not differ significantly from controls on the anxiety or depression measures of the Hospital Anxiety and Depression (HAD) scale.

Given that ALF can be seen on the day of encoding, it is natural to ask what the time course of ALF looks like and how quickly it emerges. McGibbon and Jansari (2013) recently found that ALF became apparent in a patient with temporal lobe epilepsy within 55 min. However, this relatively early forgetting is not necessarily related to ALF; Muhlert et al. (2010) reported deficits at 30 min in TEA patients, but while retention over 30 min correlated with retention over 24 h in healthy controls, this was not the case in the patients. Further work with these patients may improve our understanding of the process of memory consolidation.

In summary, ALF is not caused by a deficit in sleep-dependent memory consolidation, and ALF can be detected across an interval as short as 12 h in patients with TEA. Further work will be needed to elucidate the neural basis of this memory impairment. It may be that memory consolidation in patients with ALF is hampered by abnormalities in structural and/or functional connectivity, but it remains possible that ALF is a result of subtly abnormal processing at the stage of encoding.

## Figures and Tables

**Table 1:** TEA patient information.

Gender	Age	Age at onset	Evidence for a diagnosis of epilepsy			MRI
			EEG	Other features	Treatment response	
M	70	66	Non-specific bilateral temporal lobe slowing	Oroalimentary automatisms, gustatory hallucinations	Complete	Normal
M	62	54	L temporal epileptiform	Oroalimentary automatisms	Complete	Normal
M	68	65	Non-specific L temporal slowing	No	Complete	Normal
M	72	71	Non-specific R temporal slowing	Déjà vu	Complete	Bilateral high T2 signal in hippocampus
M	68	62	Bilateral (L more marked) mid-anterior temporal sharp and slow-wave epileptiform	Oroalimentary automatisms; brief unresponsiveness	Complete	Normal
M	76	65	Non-specific bilateral temporal slowing	Olfactory hallucinations	Complete	Normal
M	66	56	Normal	Olfactory hallucinations; brief unresponsiveness	Complete	Normal
M	64	61	Bilateral temporal epileptiform	Olfactory hallucinations; oroalimentary automatisms	Complete	Normal
M	63	59	Normal	No	Complete	Normal
M	76	73	Normal	Oroalimentary automatisms; brief unresponsiveness	Complete	Normal
F	60	55	Non-specific bilateral temporal slowing	Brief unresponsiveness	Complete	Normal

**Table 2:** Participant information. Means with SEMs in brackets. Patients and controls did not significantly differ in terms of age, years of full-time education or test scores (*p*s > .05).

	TEA patients	Controls
*N*	11	12
Gender	One female	Five females
Duration of epilepsy (months)	69.55 (±10.45)	n/a

Age	67.73 (±1.63)	63.50 (±1.44)
Years of full-time education	13.45 (±2.84)	13.04 (±3.44)

*Verbal IQ tests*		
National Adult Reading Test (NART)^a^ errors (50 words in test)	12.09 (±2.07)	11.17 (±2.06)
Predicted WAIS^b^ verbal IQ from NART errors	117.91 (±1.89)	118.67 (±1.87)
WASI^c^ vocabulary raw score (max 80)	68.27 (±6.08)	70.83 (±1.85)
WASI similarities raw score (max 48)	37.73 (±1.04)	39.67 (±1.06)
WASI verbal IQ	116.27 (±2.22)	120.83 (±3.05)

*Performance IQ tests*		
WASI block design raw score (max 71)	45.09 (±9.86)	45.92 (±3.55)
WASI matrix reasoning raw score (max 42)	26.00 (±1.03)	25.17 (±1.15)
WASI performance IQ	120.18 (±2.73)	117.00 (±3.30)

WASI full scale-4 subtests IQ	120.36 (±1.97)	121.42 (±3.01)

*Anterograde memory*		
WMS-III^d^ logical memory story: immediate recall (max 25)	14.73 (±.82)	17.50 (±1.31)
WMS-III logical memory story: delayed recall (30 min) (max 25)	12.18 (±1.30)	14.75 (±1.41)
WMS-III logical memory story: delayed recognition (30 min) (max 15)	13.18 (±.38)	13.00 (±.41)
Rey–Osterrieth complex figure^e^: copy (max 36)	33.50 (±.93)	32.38 (±.51)
Rey–Osterrieth complex figure: delayed recall (30 min) (max 36)	16.86 (±1.62)	18.25 (±1.08)
Recognition Memory Test (RMT)^f^: Words (max 50)	46.36 (±.81)	47.50 (±.87)
RMT: Faces (max 50)	41.72 (±1.40)	44.83 (±.81)

*Semantic memory*		
Graded Naming Test (GNT)^g^ (max 30)	24.27 (±1.18)	25.08 (±.63)

*Executive function*		
DKEFS^h^ verbal fluency letters (No. of words in 1 min)	46.73 (±2.76)	48.75 (±4.74)
DKEFS verbal fluency categories (No. of words in 1 min)	39.45 (±3.00)	46.33 (±2.76)
DKEFS verbal fluency switching (No. of words in 1 min)	13.45 (±.73)	15.75 (±.92)
DKEFS trails 1 (visual scanning) (seconds to complete)	28.71 (±6.13)	28.50 (±2.59)
DKEFS trails 2 (number sequencing) (seconds to complete)	40.85 (±5.04)	37.92 (±4.14)
DKEFS trails 3 (letter sequencing) (seconds to complete)	39.36 (±4.27)	38.50 (±4.02)
DKEFS trails 4 (switching) (seconds to complete)	87.57 (±9.99)	84.38 (±12.99)
DKEFS trails 5 (motor speed) (seconds to complete)	25.93 (±3.23)	24.42 (±1.95)
WMS-III Digit span forwards (max 16)	12.36 (±.79)	11.00 (±.65)
WMS-III Digit span backwards (max 14)	9.27 (±1.05)	7.67 (±.76)

*Anxiety and depression scores*		
Hospital Anxiety and Depression Scale (HADS)^i^ anxiety (max 21)	6.36 (±1.19)	5.33 (±.71)
HADS depression (max 21)	4.36 (±.87)	2.58 (±.80)

a NART (Nelson, 1982, pp. 1–13; Nelson & Willison, 1991).

b WAIS = Wechsler Abbreviated Intelligence Scale (Wechsler, 1955).

c WASI = Wechsler Abbreviated Scale of Intelligence (Wechsler, 1999).

d WMS-III = Wechsler Memory Scale-III (Wechsler, 1997).

e Rey–Osterrieth complex figure (Rey, 1941).

f RMT (Warrington, 1984).

g GNT (McKenna & Warrington, 1980).

h DKEFS = Delis–Kaplan Executive Function System (Delis, Kaplan, & Kramer, 2001).

i HADS (Zigmond & Snaith, 1983).

**Table 3:** Performance on the word-pair associates task. Means with SEMs in brackets. The three A–B word-pair tests that were used to look at memory retention (and which are plotted in Fig. 3) are in boldface.

	Controls		Patients	
	Sleep	Wake	Sleep	Wake
1st training test score (/30) (A–B)	10.17 (±2.39)	10.75 (±1.75)	8.45 (±1.83)	7.91 (±1.21)
No. trials to criterion (A–B)	2.00 (±.21)	2.50 (.26)	2.82 (±.48)	2.27 (±.14)

**Final training test score (/30)** (A–B)	**21.92** (**±.68)**	**23.17** (**±1.01)**	**22.27** (**±.75)**	**21.72** (**±.91)**
**30 min test score (/30)** (A–B)	**20.50** (**±.89)**	**22.17** (**±.95)**	**20.55** (**±.91)**	**19.36** (**±1.06)**

Immediate interference pair score (/30) (A–C)	8.50 (±2.04)	9.67 (±1.94)	10.00 (±1.68)	8.36 (±1.01)
**12 h test score (/30)** (A–B)	**18.17** (**±.98)**	**16.83** (**±1.36)**	**15.64** (**±1.15)**	**11.45** (**±.98)**
Interference pair score (/30) (A–C)	8.17 (±1.85)	9.42 (±1.94)	7.91 (±1.68)	8.09 (±1.25)

## References

[bib1] Alvarez P., Squire L.R. (1994). Memory consolidation and the medial temporal lobe: a simple neural network model. Proceedings of the National Academy of Sciences of the United States of America.

[bib2] Aly M., Moscovitch M. (2010). The effects of sleep on episodic memory in older and younger adults. Memory.

[bib3] Backhaus J., Born J., Hoeckesfeld R., Fokuhl S., Hohagen F., Jung-hanns K. (2007). Midlife decline in declarative memory consolidation is correlated with a decline in slow wave sleep. Learning and Memory.

[bib4] Bartsch T., Butler C. (2013). Transient amnesic syndromes. Nature Reviews Neurology.

[bib5] Battaglia F.P., Benchenane K., Sirota A., Pennartz C.M.A., Wiener S.I. (2011). The hippocampus: hub of brain network communication for memory. Trends in Cognitive Sciences.

[bib6] Bazil C. (2000). Sleep and epilepsy. Current Opinion in Neurology.

[bib7] Bazil C.W., Walczak T.S. (1997). Effects of sleep and sleep stage on epileptic and nonepileptic seizures. Epilepsia.

[bib8] Bell B.D., Giovagnoli A.R. (2007). Recent innovative studies of memory in temporal lobe epilepsy. Neuropsychology Review.

[bib9] Butler C.R., Bhaduri A., Acosta-Cabronero J., Nestor P.J., Kapur N., Graham K.S. (2009). Transient epileptic amnesia: regional brain atrophy and its relationship to memory deficits. Brain.

[bib10] Butler C.R., van Erp W., Bhaduri A., Hammers A., Heckemann R., Zeman A. (2013). Magnetic resonance volumetry reveals focal brain atrophy in transient epileptic amnesia. Epilepsy & Behavior.

[bib11] Butler C.R., Graham K.S., Hodges J.R., Kapur N., Wardlaw J.M., Zeman A.Z.J. (2007). The syndrome of transient epileptic amnesia. Annals of Neurology.

[bib12] Butler C.R., Zeman A.Z. (2008). Recent insights into the impairment of memory in epilepsy: transient epileptic amnesia, accelerated long-term forgetting and remote memory impairment. Brain.

[bib13] Butler C.R., Zeman A. (2008). A case of transient epileptic amnesia with radiological localization. Nature Clinical Practice Neurology.

[bib14] Coltheart M. (1981). The MRC psycholinguistic database. The Quarterly Journal of Experimental Psychology Section A: Human Experimental Psychology.

[bib15] Coughlan A.K., Oddy M.J., Crawford J.R. (2007). BIRT Memory and Information Processing Battery (BMIPB).

[bib16] Deak M.C., Stickgold R., Pietras A.C., Nelson A.P., Bubrick E.J. (2011). The role of sleep in forgetting in temporal lobe epilepsy: a pilot study. Epilepsy & Behavior.

[bib17] Delis D.C., Kaplan E., Kramer J.H. (2001). Delis-Kaplan Executive Function System (D-KEFS).

[bib18] Derrt C.P., Duncan S. (2013). Sleep and epilepsy. Epilepsy & Behavior.

[bib19] Dewar M., Garcia Y.F., Cowan N., Sala S.D. (2009). Delaying interference enhances memory consolidation in amnesic patients. Neuropsychology.

[bib20] Diekelmann S., Born J. (2010). The memory function of sleep. Nature Reviews Neuroscience.

[bib21] Diekelmann S., Büchel C., Born J., Rasch B. (2011). Labile or stable: opposing consequences for memory when reactivated during waking and sleep. Nature Neuroscience.

[bib22] Ellenbogen J.M., Hulbert J., Jiang Y., Stickgold R. (2009). The sleeping brain's influence on verbal memory: boosting resistance to interference. PLoS One.

[bib23] Ellenbogen J.M., Hulbert J.C., Stickgold R., Dinges D.F., Thompson-Schill S.L. (2006). Interfering with theories of sleep and memory: sleep, declarative memory, and associative interference. Current Biology.

[bib24] Fitzgerald Z., Thayer Z., Mohamed A., Miller L.A. (2013). Examining factors related to accelerated long-term forgetting in epilepsy using ambulant EEG monitoring. Epilepsia.

[bib25] Gallassi R. (2006). Epileptic amnesic syndrome: an update and further considerations. Epilepsia.

[bib26] Gallassi R., Morreale A., Lorusso S., Pazzaglia P., Lugaresi E. (1988). Epilepsy presenting as memory disturbances. Epilepsia.

[bib27] Girardeau G., Benchenane K., Wiener S.I., Buzsaki G., Zugaro M.B. (2009). Selective suppression of hippocampal ripples impairs spatial memory. Nature Neuroscience.

[bib28] Goncharova I.I., Zaveri H.P., Duckrow R.B., Novotny E.J., Spencer S.S. (2009). Spatial distribution of intracranially recorded spikes in medial and lateral temporal epilepsies. Epilepsia.

[bib29] Hoefeijzers S., Dewar M., Della Sala S., Zeman A., Butler C. (2013). Accelerated long-term forgetting in transient epileptic amnesia: an acquisition or consolidation deficit?. Neuropsychologia.

[bib30] Holmes G.L., Lenck-Santinin P.P. (2006). Role of interictal epileptiform abnormalities in cognitive impairment. Epilepsy & Behavior.

[bib31] Jackson O., Schacter D.L. (2004). Encoding activity in anterior medial temporal lobe supports subsequent associative recognition. NeuroImage.

[bib32] Jansari A.S., Davis K., McGibbon T., Firminger S., Kapur N. (2010). When “longterm memory” no longer means “forever”: analysis of accelerated long-term forgetting in a patient with temporal lobe epilepsy. Neuropsychologia.

[bib33] Ji D., Wilson M.A. (2007). Coordinated memory replay in the visual cortex and hippocampus during sleep. Nature Neuroscience.

[bib34] Kapur N., Markowitsch H.J. (1990). Transient epileptic amnesia: a clinically distinct form of neurological memory disorder. Transient global amnesia and related disorders.

[bib35] Kapur N., Millar J., Colbourn C., Abbott P., Kennedy P., Docherty T. (1997). Very long-term amnesia in association with temporal lobe epilepsy: evidence for multiple-stage consolidation processes. Brain and Cognition.

[bib36] Kotagal P. (2001). The relationship between sleep and epilepsy. Seminars in Pediatric Neurology.

[bib37] Lewis P., Kopelman M.D. (1998). Forgetting rates in neuropsychiatric disorders. The Journal of Neurology, Neurosurgery, and Psychiatry.

[bib38] Malmgren K., Thom M. (2012). Hippocampal sclerosis—origins and imaging. Epilepsia.

[bib39] Martin R.C., Loring D., Meador K., Lee G., Thrash N., Arena J. (1991). Impaired long-term retention despite normal auditory learning in patients with temporal lobe dysfunction. Neuropsychology.

[bib40] Matos G., Andersen M.L., do Valle A.C., Tufik S. (2010). The relationship between sleep and epilepsy: evidence from clinical trials and animal models. Journal of the Neurological Sciences.

[bib41] Mayanagi Y. (1977). The influence of natural sleep on focal spiking in experimental temporal lobe epilepsy in the monkey. Electroencephalography and Clinical Neurophysiology.

[bib42] McGibbon T., Jansari A.S. (2013). Detecting the onset of accelerated long-term forgetting: evidence from temporal lobe epilepsy. Neuropsychologia.

[bib43] McKenna P., Warrington E.K. (1980). Testing for nominal dysphasia. The Journal of Neurology, Neurosurgery, and Psychiatry.

[bib44] Mednick S., Cai D., Shuman T., Anagnostaras S., Wixted J. (2011). An opportunistic theory of cellular and systems consolidation. Trends in Neurosciences.

[bib45] Mednick S.C., Makovski T., Cai D.J., Jiang Y.V. (2009). Sleep and rest facilitate implicit memory in a visual search task. Vision Research.

[bib46] Muhlert N., Grunewald R.A., Hunkin N.M., Reuber M., Howell S., Reynders H. (2011). Accelerated long-term forgetting in temporal lobe but not idiopathic generalised epilepsy. Neuropsychologia.

[bib47] Muhlert N., Milton F., Butler C.R., Zeman A.Z. (2010). Accelerated forgetting of real-life events in Transient Epileptic Amnesia. Neuropsychologia.

[bib48] Nadel L., Moscovitch M. (1997). Memory consolidation, retrograde amnesia and the hippocampal complex. Current Opinion in Neurobiology.

[bib49] Nazer F., Dickson C.T. (2009). Slow oscillation state facilitates epileptiform events in the hippocampus. Journal of Neurophysiology.

[bib50] Nelson H.E. (1982). The National Adult Reading Test (NART): Test manual.

[bib51] Nelson H.E., Willison J. (1991). National Adult Reading Test (NART): Test manual (2nd ed.).

[bib52] Peigneux P., Laureys S., Fuchs S., Collette F., Perrin F., Reggers J. (2004). Are spatial memories strengthened in the human hippocampus during slow wave sleep?. Neuron.

[bib53] Peters K.R., Ray L., Smith V., Smith C. (2008). Changes in the density of stage 2 sleep spindles following motor learning in young and older adults. Journal of Sleep Research.

[bib54] Rasch B., Büchel C., Gais S., Born J. (2007). Odor cues during slow-wave sleep prompt declarative memory consolidation. Science.

[bib55] Rey A. (1941). L'examen psychologique dans les cas d'encephalopathie traumatique. Archives of Psychology.

[bib56] Romcy-Pereira R.N., Leite J.P., Garcia-Cairasco N. (2009). Synaptic plasticity along the sleep-wake cycle: implications for epilepsy. Epilepsy & Behavior.

[bib57] Rossi G.F., Colicchio G., Pola P. (1984). Interictal epileptic activity during sleep: a stereo-EEG study in patients with partial epilepsy. Electroencephalography and Clinical Neurophysiology.

[bib58] Rudoy J.D., Voss J.L., Westerberg C.E., Paller K.A. (2009). Strengthening individual memories by reactivating them during sleep. Science.

[bib59] Sammaritano M., Gigli G.L., Gotman J. (1991). Interictal spiking during wakefulness and sleep and the localization of foci in temporal lobe epilepsy. Neurology.

[bib60] Shatskikh T.N., Raghavendra M., Zhao Q., Cui Z., Holmes G.L. (2006). Electrical induction of spikes in the hippocampus impairs recognition capacity and spatial memory in rats. Epilepsy & Behavior.

[bib61] Spencer R.M., Gouw A.M., Ivry R.B. (2007). Age-related decline of sleep-dependent consolidation. Learning and Memory.

[bib62] Squire L.R., Zola-Morgan S. (1991). The medial temporal lobe memory system. Science.

[bib63] Stickgold R., Walker M.P. (2007). Sleep-dependent memory consolidation and reconsolidation. Sleep Medicine.

[bib64] Sud S., Sadaka Y., Massicotte C., Smith M.L., Bradbury L., Go C. (2014). Memory consolidation in children with epilepsy: does sleep matter?. Epilepsy & Behavior.

[bib65] Tambini A., Ketz N., Davachi L. (2010). Enhanced brain correlations during rest are related to memory for recent experiences. Neuron.

[bib66] Tramoni E., Felician O., Barbeau E.J., Guedj E., Guye M., Bartolomei F. (2011). Long-term consolidation of declarative memory: insight from temporal lobe epilepsy. Brain.

[bib67] Tucker M., McKinley S., Stickgold R. (2011). Sleep optimizes motor skill in older adults. Journal of the American Geriatrics Society.

[bib69] Urbain C., Di Vincenzo T., Peigneux P., Van Bogaert P. (2011). Is sleep-related consolidation impaired in focal idiopathic epilepsies of childhood? A pilot study. Epilepsy & Behavior.

[bib70] Warrington E.K. (1984). The recognition memory test.

[bib71] Wechsler D. (1955). Manual for the Wechsler adult intelligence scale.

[bib72] Wechsler D. (1997). Wechsler memory scale III.

[bib73] Wechsler D. (1999). Wechsler abbreviated scale of intelligence.

[bib74] Wilson J.K., Baran B., Pace-Schott E.F., Ivry R.B., Spencer R.M.C. (2012). Sleep modulates word-pair learning but not motor sequence learning in healthy older adults. Neurobiology of Aging.

[bib75] Wilson M.A., McNaughton B.L. (1994). Reactivation of hippocampal ensemble memories during sleep. Science.

[bib76] Zeman A.Z.J., Boniface S.J., Hodges J.R. (1998). Transient epileptic amnesia: a description of the clinical and neuropsychological features in 10 cases and a review of the literature. The Journal of Neurology, Neurosurgery, and Psychiatry.

[bib77] Zeman A.Z., Butler C.R. (2010). Transient epileptic amnesia. Current Opinion in Neurology.

[bib78] Zeman A.Z., Butler C.B., Muhlert N., Milton F. (2013). Novel forms of forgetting in temporal lobe epilepsy. Epilepsy & Behavior.

[bib79] Zigmond A.S., Snaith R.P. (1983). The hospital anxiety and depression scale. Acta Psychiatrica Scandinavica.

