# Supplementary material for: Sleep-dependent memory consolidation and accelerated forgetting
Source: Cortex. 2014 May;54(100):92–105. doi: 10.1016/j.cortex.2014.02.009 (PMC4007033; doi:10.1016/j.cortex.2014.02.009)
Supplement: Supplementary file 1 [file mmc1.docx]

**Supplementary Material**

Procedure for training and testing

During training, each pair of words was presented (A above B) in lower case, in a white Times New Roman font of size 75 (letters up to 1.6cm in height), against a black background for seven seconds. During training and testing, the 30 pairs were presented in random order. The participants sat at approximately 65cm from the screen and were asked to keep their eyes on the screen at all times.

The participants were instructed to try to remember the words as pairs and were informed about the upcoming test sessions, but were asked to avoid deliberately rehearsing the pairs in the intervals. In order to standardise encoding strategies to the extent possible and to ensure that participants had processed the target words, the participants were instructed to produce a sentence containing the two words (and where possible, no other nouns) and to say this aloud. After the seven-second presentation of each word-pair, a white fixation cross appeared. Once the sentence was complete, the experimenter, who sat behind the participants with a silent computer mouse, pressed a button. If the experimenter’s button-press occurred after more than two seconds of fixation, it caused the programme to move onto the next pair immediately. If the experimenter’s button-press occurred prior to this stage in the trial, the fixation cross was presented for two seconds between word-pairs.

After all of the pairs had been presented, there was a cued recall test. The cue words were individually displayed in isolation (with a question mark beneath) and the participants were asked to recall the paired associate. The participants were required to say their answer aloud (or pass) and then press the down arrow key to indicate that they had given their final answer. The cue word was presented for three seconds. If the down arrow key had not been pressed within this time, the question mark remained on the screen until it was pressed. The white fixation cross then appeared on the screen. During fixation, the experimenter used the silent mouse to record the correctness of the participant’s response. There was an additional 1, 2 or 3 seconds of fixation (randomly selected on each trial) after the experimenter’s response before the next cue word appeared.

If the participants did not reach the 60% criterion on the 30 pairs, they were shown each of the full pairs again, this time for five seconds each. There was a border around the pairs that was green or red depending on the correctness of the latest response for this pair. The participants were asked to re-produce their original sentence upon seeing each pair. They were instructed that if they could not recall their sentence they must produce a new one. After the five-second presentation of each word-pair, a white fixation cross appeared. Once the sentence was complete, the experimenter pressed a button on the silent mouse. If the experimenter’s button-press occurred after more than two seconds of fixation, it caused the programme to move onto the next pair immediately. If the experimenter’s button-press occurred prior to this stage in the trial, the fixation cross was presented for two seconds between word-pairs. After all the pairs had been presented, the participants were re-tested on the word-pairs and so on until they scored at least 60% in a test.

A serial reaction time task (SRTT) was performed in the 30 minutes interval between the final training test and the 30mins test. In the SRTT, the participants had to respond as quickly as possible with the appropriate keyboard button to a square flashing up on the screen at one of four possible locations. The SRTT served to standardise activity in the retention interval. Data from the SRTT are not presented here. The procedure for the 30mins A-B pair test was identical to that used in the cued recall phases of the training session.

Twelve hours after the beginning of A-B training, participants were trained on A-C word-pairs. The procedure was identical to the first training trial and first (immediate) cued recall test of A-B pair training. There was then a ten minute interval, during which the participants performed additional trials of the SRTT and a short alertness test, before the 12hrs A-B&C pair test. In this test, each cue word was presented alone (with two question marks beneath it). The participants were required to say aloud the two nouns that had previously been paired with the cue word (or pass for one or both of them) and then press the down arrow key to indicate that they had given their final answers. The cue word was presented for three seconds. If the down arrow key had not been pressed within this time, the question marks remained on the screen until it was pressed. A blue fixation cross then appeared. The experimenter indicated the correctness of the participant’s answers to the computer and 1, 2 or 3 seconds of fixation later (randomly selected), the participants were asked (by the word “When?” appearing on the screen) to indicate when they had learnt each of the paired associates they provided. They pressed the down arrow key once they had given their final answer. The experimenter indicated their correctness to the computer and 1, 2 or 3 seconds of fixation later (randomly selected), the participants were asked (with the visual prompt “Sentences?”) to try to reproduce the sentence they had used to learn each of the paired associates. When the participants had given their final answers, they pressed the down arrow key once more. The white fixation cross was then presented for three seconds before the next cue word appeared.
